# Differences in elementary-age children’s accelerometer - measured physical activity between school and summer: three-year findings from the What’s UP (Undermining Prevention) with summer observational cohort study

**DOI:** 10.1186/s12966-024-01637-z

**Published:** 2024-08-06

**Authors:** Michael W. Beets, Sarah Burkart, Christopher Pfledderer, Elizabeth Adams, R. Glenn Weaver, Bridget Armstrong, Keith Brazendale, Xuanxuan Zhu, Alexander McLain, Brie Turner-McGrievy, Russell Pate, Andrew Kaczynski, Amanda Fairchild, Brian Saelens, Hannah Parker

**Affiliations:** 1 Arnold School of Public Health, University of South Carolina, Columbia, SC USA; 2 UTHealth Houston, School of Public Health, Austin, TX USA; 3 Department of Health Sciences, University of Central Florida, Orlando, FL USA; 4 College of Arts and Sciences, University of South Carolina, Columbia, SC USA; 5 Seattle Children’s Hospital, Seattle, USA

**Keywords:** Accelerometry, Vacation, Children, Poverty

## Abstract

**Background:**

Among elementary-aged children (5-12yrs), summer vacation is associated with accelerated gains in Body Mass Index (BMI). A key behavioral driver of BMI gain is a lack of physical activity (PA). Previous studies indicate PA decreases during summer, compared to the school year but whether this difference is consistent among boys and girls, across age, and by income status remains unclear. This study examined differences in school and summer movement behaviors in a diverse cohort of children across three years.

**Methods:**

Children (N = 1024, age range 5–14 years, 48% girls) wore wrist-placed accelerometers for a 14-day wear-period during school (April/May) and summer (July) in 2021 to 2023, for a total of 6 timepoints. Mixed-effects models examined changes in school vs. summer movement behaviors (moderate-to-vigorous physical activity [MVPA], sedentary) for boys and girls, separately, and by age and household income groups (low, middle, and upper based on income-to-poverty ratio).

**Results:**

Children provided a total of 35,435 valid days of accelerometry. Overall, boys (+ 9.1 min/day, 95CI 8.1 to 10.2) and girls (+ 6.2 min/day, 95CI 5.4 to 7.0) accumulated more MVPA during school compared to summer. Boys accumulated less time sedentary (-9.9 min/day, 95CI -13.0 to -6.9) during school, while there was no difference in sedentary time (-2.7 min/day, 95CI -5.7 to 0.4) for girls. Different patterns emerged across ages and income groups. Accumulation of MVPA was consistently greater during school compared to summer across ages and income groups. Generally, the difference between school and summer widened with increasing age, except for girls from middle-income households. Accumulation of sedentary time was higher during school for younger children (5-9yrs), whereas for older children (10-14yrs), sedentary time was greater during summer for the middle- and upper-income groups. For boys from low-income households and girls from middle-income households, sedentary time was consistently greater during summer compared to school across ages.

**Conclusions:**

Children are less active and more sedentary during summer compared to school, which may contribute to accelerated BMI gain. However, this differs by biological sex, age, and income. These findings highlight the complex factors influencing movement behaviors between school and summer.

**Supplementary Information:**

The online version contains supplementary material available at 10.1186/s12966-024-01637-z.

## Background

Epidemiological evidence indicates children gain more excessive weight (represented by Body Mass Index z-scores, zBMI) during summer and vacation/holidays compared to when they are in school. [1–21] Two of the largest studies on accelerated summer weight gain [20, 21] demonstrate almost all increases in the prevalence of obesity occur during summer. Reasons for this unhealthy weight gain during summer are complex. Differences in weight gain between summer and school are hypothesized to be a function of changes in obesogenic behaviors. [22] Physical activity (PA) is a critical obesogenic behavior of interest, as it relates to overall healthy weight gain among youth [23, 24] and especially weight gain occurring during summer. [22].

Systematic reviews and multi-country analyses indicate PA is lower on less-structured days, such as the weekend, compared to more structured school days among youth. [22, 25, 26] Summer time functions much like a weekend, where fewer “structured” (i.e., pre-planned, segmented, and adult-supervised compulsory environments) [22] opportunities exist, which leads to lower activity levels. [22, 26] This “Structured Days Hypothesis” (SDH) [22] is primarily based upon comparisons between weekdays when a child is in school and weekend days – with weekend days mimicking what summer may be like for youth. [22, 25, 26] During summer, the consistent and universal presence of school and its corresponding structure are removed – i.e., no recess, physical education, or transitioning between classes, contributing to children’s daily activity levels. Studies have explored differences in PA between school and summer. [27–36] These studies demonstrate activity behaviors, such as moderate-to-vigorous PA, appear to be greater during school compared to summer, whereas mixed findings are reported for time spent sedentary.

There are challenges with interpreting previous studies. [27–36] Foremost, many have limited sample sizes of children that prevents us from robustly examining differences across ages, biological sex, and income levels. Identification of whether summer has a universal or nuanced impact on a key obesogenic behavior across child and household characteristics can help refine the field’s understanding of for whom (e.g., girls vs. boys; older vs. younger), and in what contexts (e.g., living in poverty or not), children are most affected by PA shifts from school to summer. This information can then inform intervention efforts focused on the most disproportionately impacted groups. The purpose of this study is to examine differences in movement behaviors between school and summer in a large and diverse cohort of elementary-age children in the United States. According to the Structured Days Hypothesis, [22] we hypothesized movement behaviors would be more favorable (i.e., greater MVPA, less time sedentary) during school in comparison to summer. We also explored if movement behaviors differed across income groups, age, and biological sex.

## Methods

### Study design and sample

This study used data from the What’s UP (Undermining Prevention) with Summer study (National Institutes of Health R01DK116665, WUP) designed to understand summer effects on unhealthy weight gain among elementary-aged children in the United States. The design was a longitudinal observational cohort that followed elementary-aged children (5–12 years) across three years (2021, 2022, and 2023). Children were recruited from 17 elementary schools in a mid-sized metropolitan area in the southeastern United States located at 34°N. Invitations to participate in the study were provided to parents via study flyers with a QR code and distributed via school texting services (e.g., Class Dojo). The QR code was linked to an online HIPAA compliant website to provide electronic consent. Children of parents who provided online consent, then provided verbal assent during in-person assessments. Parents and children had the right to discontinue participation in the study at any time and were asked each year if they wanted to continue in the study. The reporting of this study conforms to the STROBE checklist. [37] This study was approved by the lead author’s Institutional Review Board (Pro00080382).

### Physical activity measurement

PA was measured using a wrist-placed accelerometer (Actigraph GT9X) on the non-dominant wrist for 24-hours. The Actigraph GT9X accelerometer is a triaxial research-grade accelerometer frequently used in studies measuring children’s free-living 24-hour behaviors (i.e., PA, sedentary behavior, sleep). [38, 39] Actigraph GT9X accelerometers were initialized and downloaded using Actilife software (version 6.13.4, Actigraph LLC). Accelerometers were initialized to record data at a frequency of 30 Hz to accommodate the 14-day wear protocol need for extended battery life (ActiGraph GT9X Link + ActiLife User Guide, 2020). Stop time was not used. Idle sleep mode was enabled to preserve battery life and the display was turned off to limit distractions for children while attending school.

Movement behaviors were assessed at two timepoints each year – during school (April/May, average daylight 13 h) and again during summer (July, average daylight 14 h). Children were instructed to wear the device during school and summer for 14 consecutive days during each assessment period. Devices were distributed and returned by mail. Each mailing contained a device and information regarding wear procedures (e.g., wear while awake and sleeping, waterproof). Data were downloaded and saved in raw format as GT3X files, and raw gt3x files were processed using the GGIR package (version 2.8-2) [40] in R (Version 4.1.2; R Foundation for Statistical Computing; Vienna, Austria). Time spent in PA intensity categories was determined using intensity thresholds described by Hildebrand et al. [41] A valid wear day was defined as a minimum of 16 h.

### Child and household characteristics

At the school measurement timepoint each year, parents completed an online survey via their smartphone about the demographics of their child (biological sex and age), information about the total annual household income, and the number of people (adults and children) living in their home. This information was used to calculate the ratio of poverty to income according to U.S. Federal Poverty Guidelines established by the Department of Health and Human Services. [42]

### Refreshing longitudinal cohort

To account for parents/children electing to discontinue in the study, we refreshed the sample [43–45] in the early spring (February/March), prior to school data collection in April/May each year. Refreshing the sample, by replacing students who dropped out of the study with students who had similar demographic characteristics. Refreshing is methodology consistent with other large-scale cohort studies. [44–46] Criteria of the refreshment sample was to ensure similar proportions of students were present in the study based on child sociodemographics (i.e., biological sex, race/ethnicity, household income) from the initial year of data collection in 2021 and to replace children based upon the grade as the cohort aged over time.

### Statistical analyses

Initially, descriptive means and standard deviations were calculated for all variables. For each analysis, multi-level models were used to account for multiple days nested over time (school, summer) within each child and each school. Children were included if they provided at least one valid day of accelerometry at any timepoint. [39, 47] All analyses were run separately for boys and girls. The primary contrasts were the difference in MVPA and time spent sedentary in minutes per day between school and summer. The main predictor of interest was school vs. summer as a binary variable. These models included age and the ratio of poverty to income. All models controlled for weartime. Race/ethnicity was not included as in our analytical models given this is a social construct and there were no a priori hypotheses for its inclusion in the analytical models. [48–51] Secondary contrasts were made between the three ratio of income to poverty groups (Low ≤ 2.0, Middle > 2.0 to 3.0, and High > 3.0) [52] controlling for age. Planned contrasts were made among income groups (e.g., Low vs. Middle) and the interaction between income group and school/summer. Finally, we estimated the changes in school and summer movement behaviors overall and for each income group across age. For each analysis, the assumptions of the models were checked, and no violations were found. All analyses were conducted with Stata 18.0. Missing data were handled using full information maximum likelihood models.

**Fig. 1:**
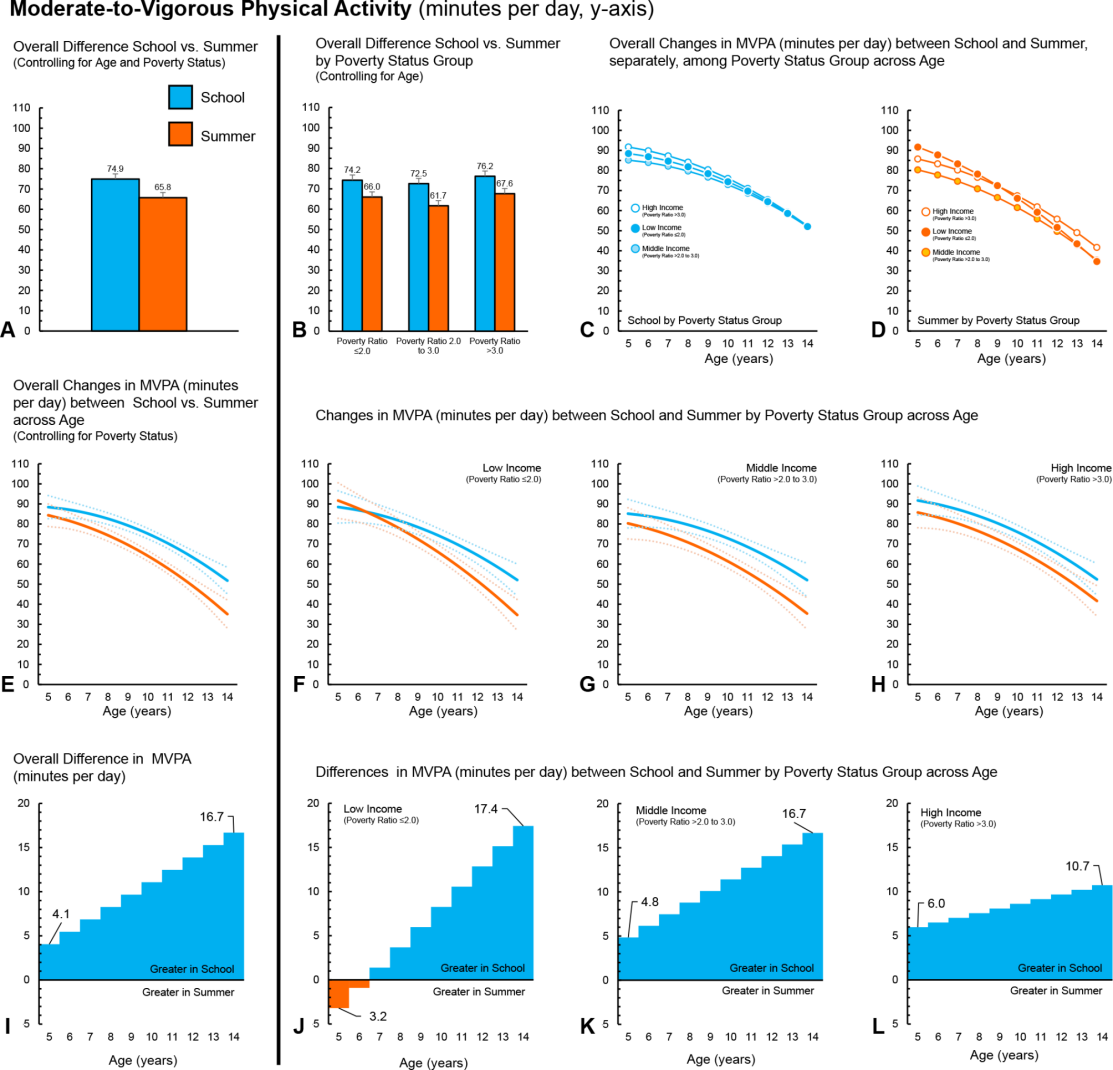
Boys (*n* = 523) – Moderate-to-Vigorous Physical Activity (minutes per day) during school and summer comparisons. (**A**) Comparison of overall average minutes per day between school and summer, controlling for age and ratio of income to poverty. (**B**) Comparison of overall average minutes per day between school and summer, controlling for age, among ratio of income to poverty groups. (**C**) Changes in minutes per day across age (years) during school among ratio of income to poverty groups. (**D**) Changes in minutes per day across age (years) during summer among ratio of income to poverty groups. (**E**) Overall changes in minutes per day across age (years) between school and summer, controlling for ratio of income to poverty (dotted lines represent 95% confidence intervals). (**F**) Changes in minutes per day across age (years) between school and summer for children in the low-income group (dotted lines represent 95% confidence intervals). (**G**) Changes in minutes per day across age (years) between school and summer for children in the middle-income group (dotted lines represent 95% confidence intervals). (**H**) Changes in minutes per day across age (years) between school and summer for children in the high-income group (dotted lines represent 95% confidence intervals). (**I**) *Overall differences, by age, in minutes per day between school and summer, controlling for ratio of income to poverty. (**J**) *Differences, by age, in minutes per day between school and summer, for children in the low-income group. (**K**) *Differences, by age, in minutes per day between school and summer, for children in the middle-income group. (**L**) *Differences, by age, in minutes per day between school and summer, for children in the high-income group. *Panels I, J, K and L ORANGE represents greater minutes per day during summer compared to school at a given age; BLUE represents greater minutes per day during school compared to summer at a given age

**Fig. 2:**
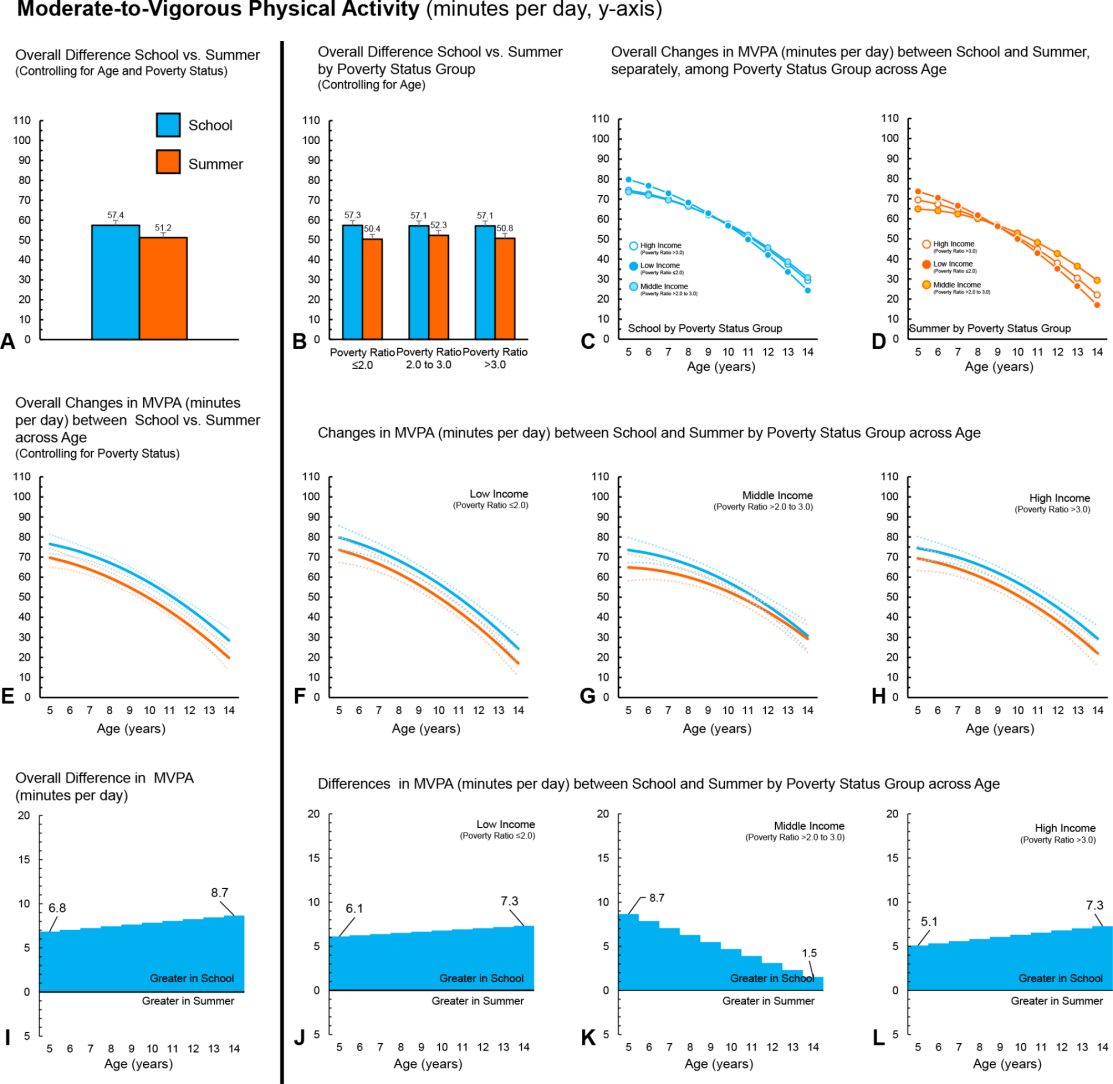
Girls (*n* = 501) – Moderate-to-Vigorous Physical Activity (minutes per day) during school and summer comparisons. (**A**) Comparison of overall average minutes per day between school and summer, controlling for age and ratio of income to poverty. (**B**) Comparison of overall average minutes per day between school and summer, controlling for age, among ratio of income to poverty groups. (**C**) Changes in minutes per day across age (years) during school among ratio of income to poverty groups. (**D**) Changes in minutes per day across age (years) during summer among ratio of income to poverty groups. (**E**) Overall changes in minutes per day across age (years) between school and summer, controlling for ratio of income to poverty (dotted lines represent 95% confidence intervals). (**F**) Changes in minutes per day across age (years) between school and summer for children in the low-income group (dotted lines represent 95% confidence intervals). (**G**) Changes in minutes per day across age (years) between school and summer for children in the middle-income group (dotted lines represent 95% confidence intervals). (**H**) Changes in minutes per day across age (years) between school and summer for children in the high-income group (dotted lines represent 95% confidence intervals). (**I**) *Overall differences, by age, in minutes per day between school and summer, controlling for ratio of income to poverty. (**J**) *Differences, by age, in minutes per day between school and summer, for children in the low-income group. (**K**) *Differences, by age, in minutes per day between school and summer, for children in the middle-income group. (**L**) *Differences, by age, in minutes per day between school and summer, for children in the high-income group. *Panels I, J, K and L ORANGE represents greater minutes per day during summer compared to school at a given age; BLUE represents greater minutes per day during school compared to summer at a given age

## Results

A total of N = 501 girls and N = 523 boys provided at least one valid accelerometer assessment across the 6 measurement timepoints. This resulted in a total of 17,001 valid days (average 29.1, range 1 to 92) from girls and 18,434 (average 29.8, range 1 to 92) valid days for boys. The sample demographics at each measurement timepoint are presented in Table 1. The number of children at each age (age rounded to the nearest year) across the timepoints are presented in Table 2. On average, boys and girls provided at least one valid day of accelerometer data for 3.4 and 3.2 timepoints (from a total of 6 possible timepoints across 3 years, see Table 3). A comparison between children who provided accelerometry data versus those who did not at each time point can be found in **Supplemental Table 1**.

**Table 1:** Demographic characteristics by measurement timepoint

			Race/Ethnicity				Age (years)					Girls		Ratio of Income to Poverty			
Year	Timepoint	Sample Size	Black	White	Multi		Mean	SD	Min	Max				Mean	SD	Min	Max
2021	School	569	33.7%	55.5%	8.0%		8.8	1.7	4.7	12.2		47.3%		2.6	1.1	0.2	4.8
	Summer	465	33.1%	55.6%	8.4%		8.9	1.7	4.8	12.4		49.3%		2.6	1.1	0.2	5.1
2022	School	571	37.2%	50.2%	8.4%		9.4	1.7	5.2	13.0		48.6%		2.3	1.1	0.3	5.2
	Summer	474	37.2%	49.6%	8.9%		9.6	1.7	5.6	13.1		47.9%		2.4	1.0	0.3	5.2
2023	School	635	33.3%	54.9%	9.2%		10.6	1.5	6.4	14.0		47.9%		2.4	1.1	0.2	5.2
	Summer	570	33.3%	54.9%	9.7%		10.7	1.6	6.5	14.1		47.4%		2.5	1.0	0.2	5.2

**Table 2:** Number of children by age (rounded whole year) at each assessment timepoint

		Age (year)										
Year	Timepoint	5	6	7	8	9	10	11	12	13	14	Total
2021	School	11	68	61	91	122	130	68	18			569
	Summer	7	39	61	72	89	101	74	22			465
2022	School	3	27	69	81	99	107	126	48	11		571
	Summer		18	49	65	84	83	104	56	15		474
2023	School		1	24	52	64	144	157	136	48	9	635
	Summer			19	45	59	110	141	123	59	14	570
	**Total**	21	153	283	406	517	675	670	403	133	23	

**Table 3:** Number of assessment periods and years children completed accelerometry measures

Assessments Present (range 1 to 6)	Boys (*n* = 523)			Girls (*n* = 501)	
Average (% and n)	3.4			3.2	
1	14.6%	90		18.3%	107
2	28.7%	177		30.2%	177
3	7.8%	47		8.6%	50
4	17.6%	109		15.8%	92
5	8.0%	49		6.6%	39
6	23.4%	145		20.5%	120
Years Present^a^ (% and n)					
2021	18.2%	112		19.7%	115
2022	4.8%	30		7.0%	41
2023	17.6%	109		19.5%	114
2021 & 2022	7.6%	47		9.2%	54
2022 & 2023	15.2%	94		13.8%	81
2021 & 2023	1.4%	9		0.6%	4
2021, 2022, 2023	35.1%	218		30.2%	177

^a^ Includes school and summer

### Overall findings – MVPA

Comparisons between MVPA accumulated daily during school and summer overall and by ratio of income to poverty groups can be found in Tables 4 and 5; Fig. 1A and B and 2A and B for boys and girls, respectively. Both boys (+ 9.1, 95CI 8.1 to 10.2) and girls (+ 6.2, 95CI 5.4 to 7.0) accumulated more MVPA minutes per day during school, compared to summer. For both boys and girls, MVPA was higher during school, compared to summer, across all three poverty to income groups. The lack of interactions by income group and school/summer provides evidence that the differences between MVPA accumulated in school and summer were similar across income groups for boys and girls.

**Table 4:** Boys (*n* = 618) – comparison of moderate-to-vigorous physical activity and time spent sedentary (minutes per day) between school and summer

	Timepoint	Est	(95CI)		Within ^a^			Between Income to Poverty Groups										
									School				Summer			Interactions ^b^		
					Diff	(95CI)		Contrast	Diff	(95CI)			Diff	(95CI)		Diff	(95CI)	
**Moderate to Vigorous Physical Activity**																		
Overall ^c^	Summer	65.8	(63.2,	68.3)	**9.1 (8.1**,** 10.2)**													
	School	74.9	(72.4,	77.4)														
**Ratio of Income to Poverty Group** ^**d**^																		
Low-Income (≤ 2.0)	Summer	66.0	(62.5,	69.5)	**8.3 (6.4**,** 10.1)**			L v M	-1.7	(-4.8,	1.4)		**− 4.3**	**(-7.5**,	**-1.1)**	NS		
	School	74.2	(70.8,	77.6)				L v H	1.9	(-1.5,	5.4)		1.6	(-1.9,	5.2)	NS		
Middle-Income (2.0 to 3.0)	Summer	61.7	(58.3,	65.0)	**10.2 (6.7**,** 13.7)**			M v H	**3.6**	**(0.8**,	**6.4)**		**5.9**	**(3.1**,	**8.8)**	NS		
	School	72.5	(69.2,	75.8)														
High-Income (> 3.0)	Summer	67.6	(64.2,	71.0)	**8.6 (7.0**,** 10.1)**													
	School	76.2	(72.8,	79.5)														
**Time Spent Sedentary**																		
Overall ^c^	Summer	499.5	(491.3,	507.6)	**-9.9 (-13.0**,** -6.9)**													
	School	489.6	(481.5,	497.6)														
**Ratio of Income to Poverty Group** ^**d**^																		
Low-Income (≤ 2.0)	Summer	502.3	(492.0,	512.6)	**-10.4 (-16.0**,** -4.8)**			L v M	-4.9	(-14.1,	4.4)		-4.1	(-13.8,	5.6)	NS		
	School	491.8	(481.8,	501.8)				L v H	-1.4	(-11.7,	8.9)		-3.2	(-13.8,	7.5)	NS		
Middle-Income (2.0 to 3.0)	Summer	498.2	(488.2,	508.1)	**-11.2 (-17.1**,** -5.3)**			M v H	3.5	(-4.9,	11.8)		0.9	(-7.6,	9.5)	NS		
	School	487.0	(477.2,	496.7)														
High-Income (> 3.0)	Summer	499.1	(489.1,	509.1)	**-8.6 (-13.3**,** -4.0)**													
	School	490.5	(480.6,	500.3)														

^**a**^ Positive within values represent greater minutes per day during school compared to summer

^b^ Income to Poverty Group – x – School vs. Summer interaction

^c^ Controlling for age (years) and ratio of income to poverty

^d^ Controlling for age (years)

**GREYED** values represent differences where the 95% confidence intervals do not include zero

*Abbreviations*: L = Low-Income; M = Middle-Income; H = High-Income

**Table 5:** Girls (*n* = 585) – comparison of moderate-to-vigorous physical activity and time spent sedentary (minutes per day) between school and summer

	Timepoint	Est	(95CI)					Between Income to Poverty Groups										
					Within ^a^				School				Summer			Interactions ^b^		
					Diff	(95CI)		Contrast	Diff	(95CI)			Diff	(95CI)		Diff	(95CI)	
**Moderate to Vigorous Physical Activity**																		
Overall ^c^	Summer	51.2	(48.8,	53.7)	**6.2 (5.4**,** 7.0)**													
	School	57.4	(55.0,	59.9)														
**Ratio of Income to Poverty Group** ^**d**^																		
Low-Income (≤ 2.0)	Summer	50.4	(47.4,	53.4)	**6.9 (5.6**,** 8.3)**			L v M	-0.2	(-2.7,	2.3)		1.9	(-0.7,	4.5)	NS		
	School	57.3	(54.3,	60.3)				L v H	-0.2	(-3.1,	2.6)		0.4	(-2.4,	3.3)	NS		
Middle-Income (2.0 to 3.0)	Summer	52.3	(49.3,	55.4)	**4.8 (3.1**,** 6.5)**			M v H	0.0	(-2.3,	2.2)		-1.5	(-3.9,	0.9)	NS		
	School	57.1	(54.1,	60.1)														
High-Income (> 3.0)	Summer	50.8	(47.8,	53.8)	**6.3 (5.0**,** 7.5)**													
	School	57.1	(54.1,	60.1)														
**Time Spent Sedentary**																		
Overall ^c^	Summer	509.7	(500.2,	519.3)	-2.7 (-5.7, 0.4)													
	School	507.1	(497.6,	516.5)														
**Ratio of Income to Poverty Group** ^**d**^																		
Low-Income (≤ 2.0)	Summer	516.6	(505.1,	528.2)	**-13.5 (-18.6**,** -8.5)**			L v M	2.9	(-6.5,	12.2)		-8.1	(-17.8,	1.7)	**20.6**	**(13.6**,	**27.5)**
	School	503.1	(491.7,	514.5)				L v H	9.8	(-0.6,	20.1)		**-10.8**	**(-21.4**,	**-0.2)**	**10.9**	**(2.7**,	**19.2)**
Middle-Income (2.0 to 3.0)	Summer	508.6	(496.9,	520.2)	-2.6 (-9.0, 3.8)			M v H	6.9	(-1.6,	15.3)		-2.7	(-11.6,	6.1)			
	School	506.0	(494.6,	517.4)														
High-Income (> 3.0)	Summer	505.8	(494.3,	517.4)	**7.0 (2.3**,** 11.8)**													
	School	512.9	(501.4,	524.3)														

^**a**^ Positive within values represent greater minutes per day during school compared to summer

^b^ Income to Poverty Group – x – School vs. Summer interaction

^c^ Controlling for age (years) and ratio of income to poverty

^d^ Controlling for age (years)

**GREYED** values represent differences where the 95% confidence intervals do not include zero

*Abbreviations*: L = Low-Income; M = Middle-Income; H = High-Income

Analysis examining changes across age showed that as children aged, MVPA declined by an average of -5.1 (95CI -5.9 to -4.4) and − 5.2 (95CI -5.8 to -4.5) minutes for each year increase in age for boys and girls, respectively (Figs. 1E and 2E). For boys, the decline in MVPA widened between school and summer as they got older, with a yearly age-x-school/summer interaction of + 1.3 (95CI 0.7 to 1.9) minutes for every year increase in age. For girls, there was no statistically significant interaction between age and school/summer (0.1, 95CI − 0.03 to 0.5). When examining declines in MVPA by poverty to income groups, boys and girls from the low-income groups’ MVPA declined by -6.5 (95CI -8.1 to -5.0) and − 6.0 (95CI -7.2 to -5.1) minutes for every year increase in age (Figs. 1F and 2F), whereas boys and girls in the middle-income group declined by -4.5 and − 4.7 min (Fig. 1G), and boys and girls in the high-income by -5.1 min for every year increase in age (Figs. 1H and 2H). Boys in the low- and middle-income groups exhibited a yearly age-x-school/summer interaction of + 2.4 (95CI 1.3 to 3.5) and + 1.5 (95CI 0.4 to 2.6) minutes for every year increase in age. No statistically significant age-x-school/summer interactions were observed for boys from the high-income group or the girls in any of the three income to poverty groups.

**Fig. 3:**
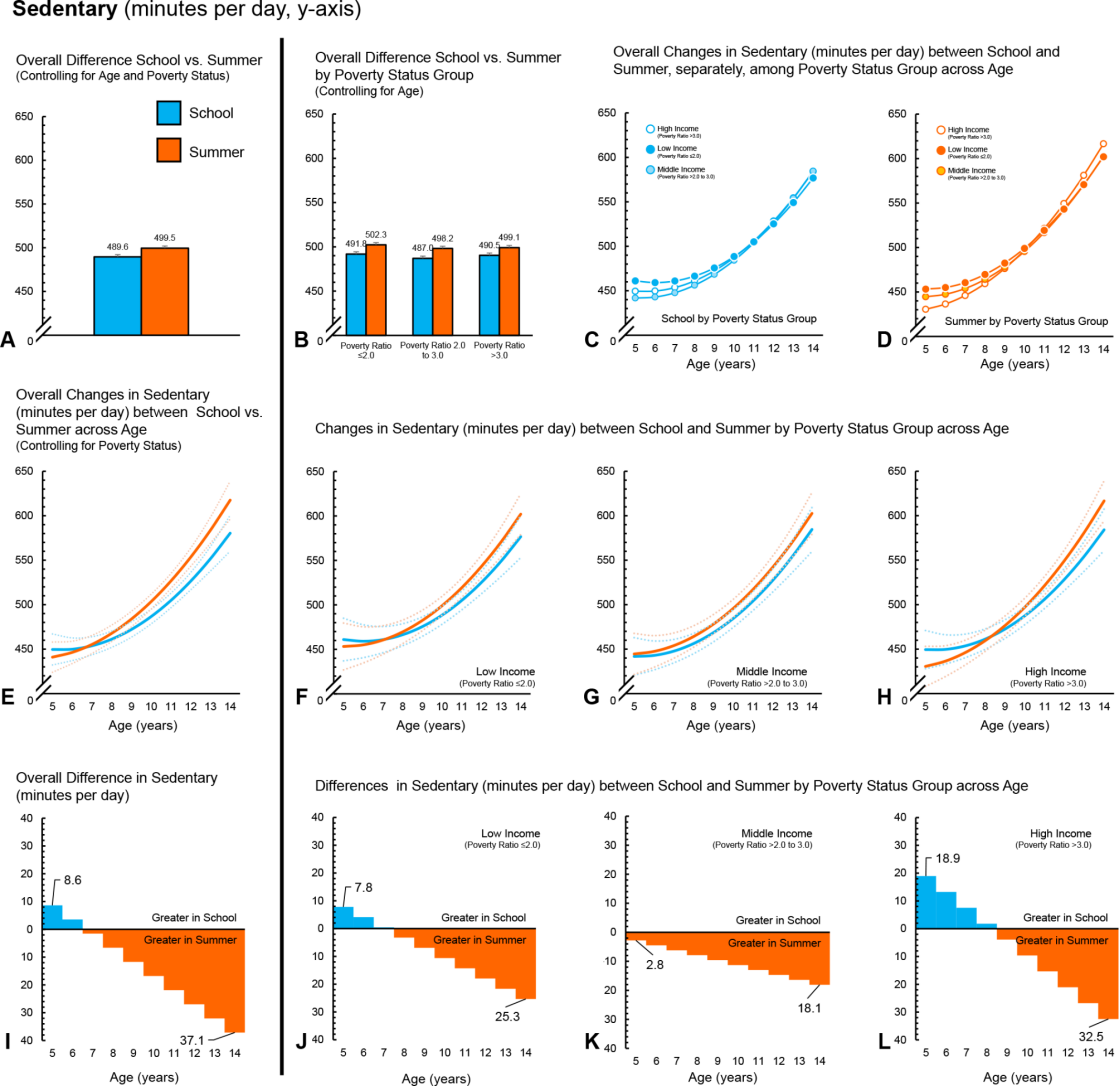
Boys (*n* = 523) – Time spent sedentary (minutes per day) during school and summer comparisons. (**A**) Comparison of overall average minutes per day between school and summer, controlling for age and ratio of income to poverty. (**B**) Comparison of overall average minutes per day between school and summer, controlling for age, among ratio of income to poverty groups. (**C**) Changes in minutes per day across age (years) during school among ratio of income to poverty groups. (**D**) Changes in minutes per day across age (years) during summer among ratio of income to poverty groups. (**E**) Overall changes in minutes per day across age (years) between school and summer, controlling for ratio of income to poverty (dotted lines represent 95% confidence intervals). (**F**) Changes in minutes per day across age (years) between school and summer for children in the low-income group (dotted lines represent 95% confidence intervals). (**G**) Changes in minutes per day across age (years) between school and summer for children in the middle-income group (dotted lines represent 95% confidence intervals). (**H**) Changes in minutes per day across age (years) between school and summer for children in the high-income group (dotted lines represent 95% confidence intervals). (**I**) *Overall differences, by age, in minutes per day between school and summer, controlling for ratio of income to poverty. (**J**) *Differences, by age, in minutes per day between school and summer, for children in the low-income group. (**K**) *Differences, by age, in minutes per day between school and summer, for children in the middle-income group. (**L**) *Differences, by age, in minutes per day between school and summer, for children in the high-income group. *Panels I, J, K and L ORANGE represents greater minutes per day during summer compared to school at a given age; BLUE represents greater minutes per day during school compared to summer at a given age

### Overall findings – time spent sedentary

**Fig. 4:**
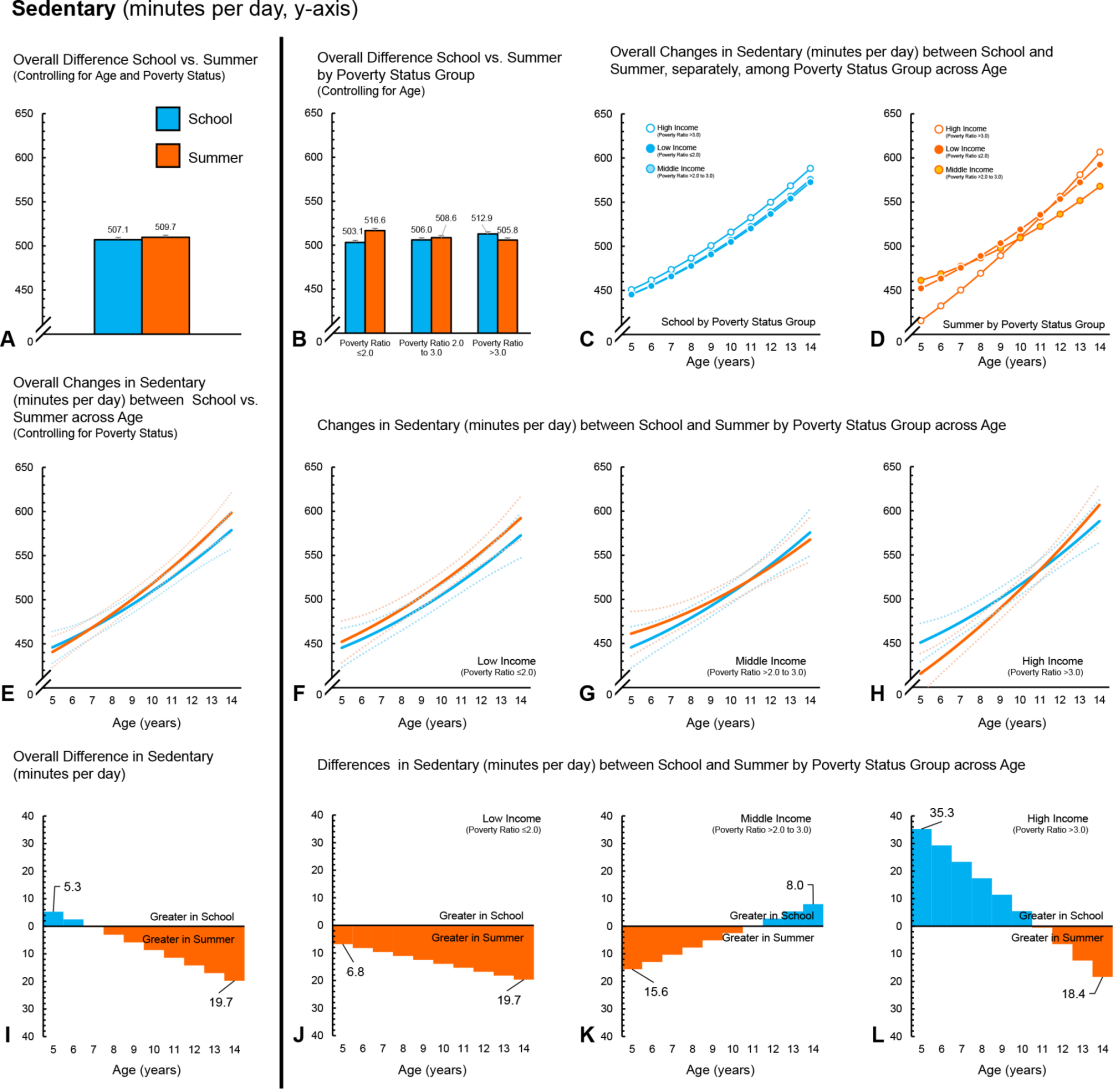
Girls (*n* = 501) – Time spent sedentary (minutes per day) during school and summer comparisons. (**A**) Comparison of overall average minutes per day between school and summer, controlling for age and ratio of income to poverty. (**B**) Comparison of overall average minutes per day between school and summer, controlling for age, among ratio of income to poverty groups. (**C**) Changes in minutes per day across age (years) during school among ratio of income to poverty groups. (**D**) Changes in minutes per day across age (years) during summer among ratio of income to poverty groups. (**E**) Overall changes in minutes per day across age (years) between school and summer, controlling for ratio of income to poverty (dotted lines represent 95% confidence intervals). (**F**) Changes in minutes per day across age (years) between school and summer for children in the low-income group (dotted lines represent 95% confidence intervals). (**G**) Changes in minutes per day across age (years) between school and summer for children in the middle-income group (dotted lines represent 95% confidence intervals). (**H**) Changes in minutes per day across age (years) between school and summer for children in the high-income group (dotted lines represent 95% confidence intervals). (**I**) *Overall differences, by age, in minutes per day between school and summer, controlling for ratio of income to poverty. (**J**) *Differences, by age, in minutes per day between school and summer, for children in the low-income group. (**K**) *Differences, by age, in minutes per day between school and summer, for children in the middle-income group. (**L**) *Differences, by age, in minutes per day between school and summer, for children in the high-income group. *Panels I, J, K and L ORANGE represents greater minutes per day during summer compared to school at a given age; BLUE represents greater minutes per day during school compared to summer at a given age

Overall, boys spent less time sedentary during school, compared to summer (-9.9, 95CI -13.0 to -6.9 min per day). In contrast, for girls, there was no overall difference between school and summer in time spent sedentary (-2.7, 95CI -5.7 to 0.4 min per day). Across all three ratios of poverty to income groups, boys accumulated less time sedentary during school than summer (Tables 4 and 5; Fig. 3A and B and 4A and B). Girls in the low-income group spent less time sedentary during school compared to summer. There was no difference in time spent sedentary between school and summer for girls in the middle-income group. Conversely, girls in the high-income group spent more time sedentary in school compared to summer. For boys, there were no differences between the poverty to income ratio groups in terms of changes in time spent sedentary for school or summer. Likewise, there were no interactions between poverty to income group and school/summer. For girls, there were no between group differences in changes in time spent sedentary for school. During summer, girls in the low-income group spent less time sedentary than girls in the high-income group. Estimates from the interactions indicated girls in the high-income group changed the amount of time spent sedentary in the summer by + 20.6 (95CI 13.6 to 27.5) and + 10.9 (95CI 2.7 to 19.2) more minutes per day compared to girls in the low-income group and middle-income groups, respectively.

Analysis examining changes across age showed that as children aged, time spent sedentary increased by an average of + 17.9 (95CI 15.6 to 20.3) and + 17.0 (95CI 14.6 to 19.3) minutes for each year increase in age for boys and girls, respectively (Figs. 3E and 4E). For boys and girls, the difference in time spent sedentary widened as they got older, with a yearly age-x-school/summer interaction of -4.6 (95CI -6.3 to -2.9) and − 2.6 (95CI -4.3 to -1.0), minutes for every year increase in age, respectively. When examining increases in time spent sedentary by poverty to income groups, sedentary time for boys and girls from the low-income groups time spent increased by + 15.8 (95CI ) and + 18.5 (95CI ) minutes for every year increase in age (Figs. 3F and 4F), whereas boys and girls in the middle-income group increased by + 19.2 (95CI ) and + 11.5 (95CI ) minutes (Figs. 3G and 4G) and boys and girls in the high-income by + 16.4 (95CI ) and + 19.8 (95CI ) minutes for every year increase in age (Figs. 3H and 4H). For boys, the low- and middle-income groups exhibited a yearly age-x-school/summer interaction of -3.7 min (95CI -6.9 to -0.4) and − 6.1 (95CI -8.7 to -3.6) for every year increase in age, respectively. Girls in the high-income group exhibited a yearly age-x-school/summer interaction of -6.3 (95CI -8.9 to -3.7). No statistically significant yearly age-x-school/summer interactions were observed for boys from the high-income group or girls from the low- or middle-income groups.

**Fig. 5:**
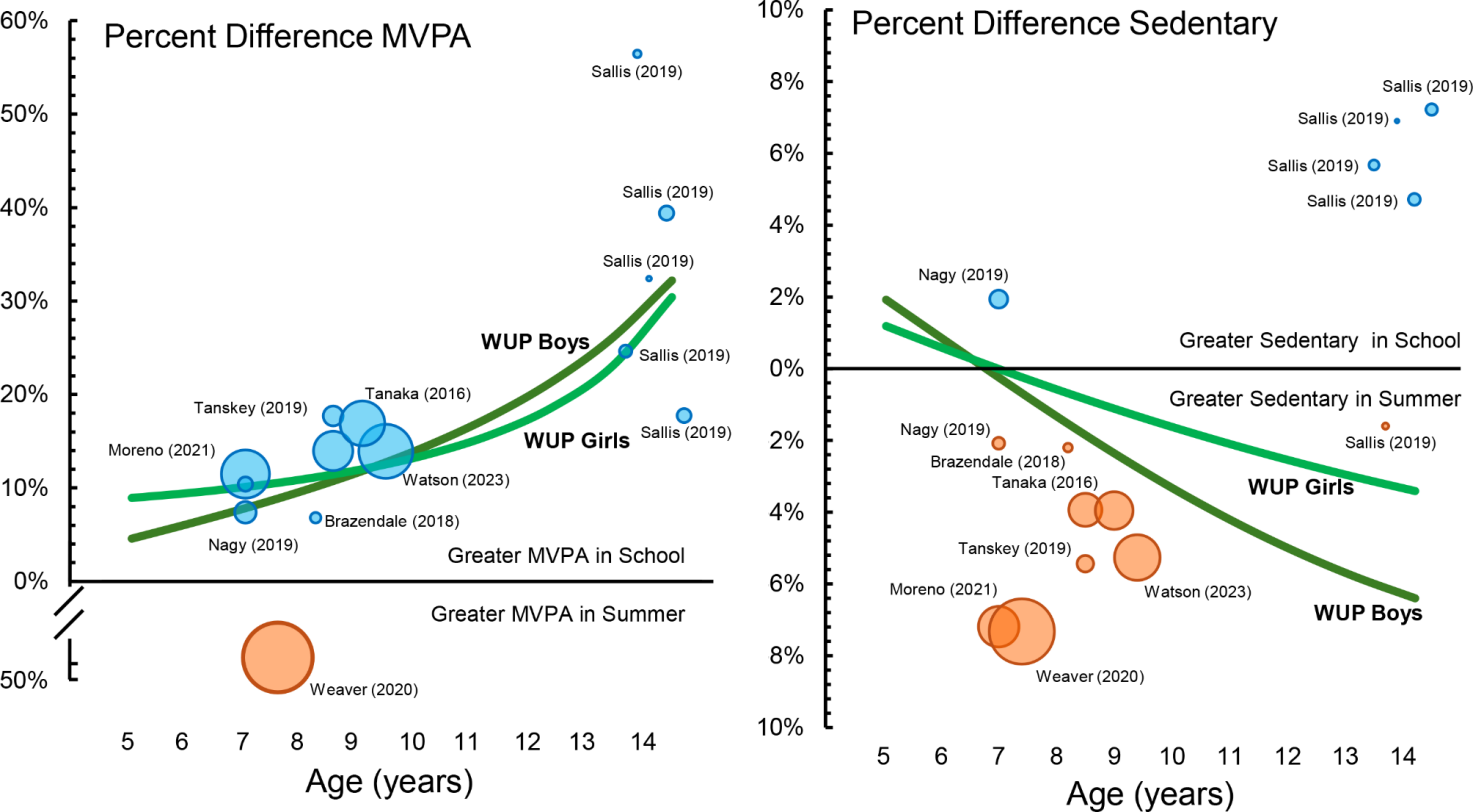
Comparison of the percent difference in time spent sedentary and moderate-to-vigorous physical activity (MVPA) across age (years) among published studies using objective measures compared to the percent differences in the current What’s UP with Summer (WUP) study. *Note*: GREEN Lines are the present difference between school and summer for the WUP study cohort among boys (Dark Green) and girls (Light Green). BLUE circles indicate greater MVPA or time spent sedentary during school. ORANGE circles indicate greater MVPA or time spent sedentary during summer. Percent difference calculated as School minus Summer divided by School. The size of each circle represents the size of the sample. Circles are scaled relative to sample size across published studies (median *N* = 52, range 16 to 188, total sample size across studies 946)

## Discussion

This study examined differences in movement behaviors when children were attending school compared to when they were on summer vacation. The findings indicate that children are generally less active and more sedentary during the summer than when they attend school. Moreover, the difference between school and summer movement behaviors widens as children get older. However, this widening effect is variable across income levels, pointing to the complex processes that influence movement behaviors in summer. This evidence, along with data from previous studies, [27–36] indicates summer is a time when movement behaviors worsen among elementary-age children, which could lead to unhealthy weight gains.

Our findings support the directionality detailed in the Structured Days Hypothesis (SDH). [22] Specifically, obesogenic behaviors, including movement behaviors, worsen during summer compared to school. The SDH posits that the key driver of this worsening is the absence or lessening of structured opportunities during the summer vs. the school year. Most children in the US attend school for ~ 7 h each day, 5 days per week. During this time, school provides several opportunities for children to engage in planned/intentional activity, such as physical education and recess, as well as incidental activity from moving to/from classrooms throughout the day. Additionally, movement behaviors can be influenced before and after school by participating in active transportation or from the need to wake up, get ready for school, and transport to/from school. In a 24-hour day, accounting for 8–9 h of sleep, [53] ~45% of waking hours are spent at school. The remaining waking hours can be considered discretionary or non-school time. In the summer, the removal of school shifts this previously structured time to discretionary time, essentially doubling the amount of “unstructured” wake time. This shift in the allocation of time from highly structured to (often) less structured is a plausible explanation for the change in movement behaviors identified in the current study. Structure as a causal factor is also supported by studies during the COVID-19 pandemic. Indeed, when examining school days vs. virtual school days, findings demonstrate when children attend school in-person, their activity behaviors are more favorable. [54] Importantly, studies [54] show older children’s activity behaviors benefit more from being in-person at school than younger children. These data [54] and the findings from the current study indicate when children attend school in-person, they accumulate more MVPA and spend less time sedentary.

An important caveat here is, the reduced PA and increase in time spent sedentary during summer does not imply there are no structured opportunities for children during the summer. Examples include summer day camp, summer sport leagues, and summer academic programs. [15, 55] But, unlike school, attending a structured opportunity during summer is not universally required nor always attainable based on income. Indeed, national estimates indicate summer day camps (one of the most common forms of summer structure) are predominantly attended by children from high-income households. [55–57] The differences in MVPA and time spent sedentary among income groups could be related to this inequity of access to summer programming. [55–57] In support of this, there is evidence that suggests a positive link between structure during the summer and movement behaviors. [9, 58, 59] Evidence from quasi-experimental studies indicates during the summer, children accumulate more MVPA on days when they attend a summer day camp, compared to days they do not. [6] These data provide further support for the SDH, and point to structure as a possible intervention to prevent summer declines in PA. Additional observational and experimental studies are needed to evaluate the association among structured program attendance and movement behaviors.

Differences between time spent sedentary during school versus summer revealed different patterns based on age. For younger children (< 9 years), time spent sedentary was, at times, greater during school compared to summer. This relationship mostly reversed with increasing age, whereby ages 10-years and older, summer was associated with a greater accumulation of time spent sedentary compared to school. These differences could be explained by classroom management rules being more restrictive for younger children, thereby increasing time spent sedentary during school. [60, 61] Conversely, as children age, they gain more autonomy. With this, they may prefer to choose more sedentary activities (e.g., watching TV, viewing smartphones) that may result in greater amounts of sedentary time during summer. Regardless of the reasons, these findings provide evidence of the ways summer differentially impacts movement behaviors across age groups. This suggests a one-size-fits-all approach to addressing PA during summer may be ineffective, and unique strategies tailored to the age of the children are necessary.

Our findings are mostly consistent with published studies using an objective measure of movement behaviors. [28–35] The comparison between the findings from our study and previous studies by others are presented in Fig. 5. The majority of published findings show an increase of ~ 2% to ~ 8% in time spent sedentary during the summer compared to school. [28–36] A notable difference is the findings from Sallis et al., [30] which shows older youth (14 years, middle school) are more sedentary during school compared to summer. The MVPA findings from WUP show a strong concurrence with all published studies, except findings from Weaver et al., [35] that children accumulate more MVPA during school compared to summer.

There are several limitations with this study. First, not all children were measured at all six time points. Nevertheless, there was a large number of children measured at multiple timepoints, thus providing a robust estimate of movement behaviors during school and summer. Second, these data come from a single geographical location, which may have local weather conditions during school and summer that are different from locales in higher or lower latitudes. Additionally, the racial/ethnic and income ratio breakdowns do not reflect the US as a whole but are representative of the southeastern US. Third, these data only include elementary-aged children, and it is unclear if these associations would be similar or different in younger (< 5 years) or older children. Finally, at several of the time points, children with accelerometry data were slightly more affluent compared to children who did not provide accelerometry data. This study also had multiple strengths, including the large number of children assessed, the large number of days evaluated during school and summer, the collection of PA and sedentary behaviors using an objective device, and the diverse (based on race and income) cohort of children included.

## Conclusions

This study provides robust evidence of consistent yearly declines in MVPA and an increase in time spent sedentary during summer, compared to the school year. Furthermore, these differences become more pronounced as children age. Results point to the importance of structured environments, like school, to help maintain healthful movement behaviors. Differences in movement behaviors were moderated by income, as well as biological sex and age, suggesting complex interactions occur during the summer that would require unique intervention strategies to address.

## Electronic supplementary material

Below is the link to the electronic supplementary material.

Supplementary Material 1

Supplementary Material 2

## Data Availability

The datasets generated and/or analyzed in the current study are not publicly available because additional analyses are ongoing. Data will be made available from the corresponding author on reasonable request at the time the primary outcome papers are published.

## Abbreviations

MVPA: moderate-to-vigorous physical activity
PA: physical activity
BMI: body mass index
Yrs: years
95CI: 95% confidence intervals
Mins/day: minutes per day
SDH: structured days hypothesis
UP: undermining prevention
WUP: What’s UP
Hrs: hours
Hz: hertz
