# Correction: Differences in elementary-age children’s accelerometer - measured physical activity between school and summer: three-year findings from the What’s UP (Undermining Prevention) with summer observational cohort study

**DOI:** 10.1186/s12966-025-01774-z

**Published:** 2025-06-02

**Authors:** Michael W. Beets, Sarah Burkart, Christopher Pfledderer, Elizabeth Adams, R. Glenn Weaver, Bridget Armstrong, Keith Brazendale, Xuanxuan Zhu, Alexander McLain, Brie Turner-McGrievy, Russell Pate, Andrew Kaczynski, Amanda Fairchild, Brian Saelens, Hannah Parker

**Affiliations:** 1 Arnold School of Public Health, University of South Carolina, Columbia, SC USA; 2 UTHealth Houston, School of Public Health, Austin, TX USA; 3 Department of Health Sciences, University of Central Florida, Orlando, FL USA; 4 College of Arts and Sciences, University of South Carolina, Columbia, SC USA; 5 Seattle Children’s Hospital, Seattle, USA

**Correction: Beets** ***et al. International Journal of Behavioral Nutrition and Physical Activity*** **(2024) 21:86**

10.1186/s12966-024-01637-z

After the publication of the Original Article, the authors reported that some of the data were incorrect. In running the descriptive statistics of the sample there was a sub-sample of children who transitioned to a new school. When tabulating these data across the entire 3 years by children and school, it double counted these children in the estimate. The sample size for boys and girls should have been *n* = 523 and *n* = 501, respectively, thus totalling 1,204, instead of *n* = 618 and *n* = 585 originally published.

However, the authors found that the errors did not influence any of their analytical model estimates.

The original article has been corrected.

