# Supplementary material for: Differences in elementary-age children’s accelerometer - measured physical activity between school and summer: three-year findings from the What’s UP (Undermining Prevention) with summer observational cohort study
Source: Int J Behav Nutr Phys Act. 2024 Aug 6;21:86. doi: 10.1186/s12966-024-01637-z (PMC11304806; doi:10.1186/s12966-024-01637-z)
Supplement: Supplementary file 2 — Supplementary Material 2 [file 12966_2024_1637_MOESM2_ESM.doc]

**Supplemental Table 1.** Comparison of demographic characteristics between children providing accelerometry data versus those children without accelerometry data at each timepoint.

|  |  | Age | | | | | |  | Ratio Poverty to Income | | | | | |  | % Girls | | | |
| --- | --- | --- | --- | --- | --- | --- | --- | --- | --- | --- | --- | --- | --- | --- | --- | --- | --- | --- | --- |
|  |  | No Accelerometry | |  | Accelerometry |  |  |  | No Accelerometry | |  | Accelerometry |  |  |  | No Accelerometry |  | Accelerometry |  |
| Year | Timepoint | M | SD |  | M | SD | P-value |  | M | SD |  | M | SD | P-value |  | % |  | % | P-value |
| 2021 | School | 8.7 | 1.7 |  | 8.8 | 1.7 | 0.513 |  | 1.9 | 1.1 |  | 2.6 | 1.1 | 0.000 |  | 53.7% |  | 47.3% | 0.187 |
|  | Summer | 8.9 | 1.7 |  | 8.9 | 1.7 | 0.761 |  | 2.1 | 1.2 |  | 2.6 | 1.1 | 0.000 |  | 53.2% |  | 49.3% | 0.191 |
| 2022 | School | 9.5 | 1.8 |  | 9.4 | 1.7 | 0.374 |  | 2.1 | 1.2 |  | 2.3 | 1.1 | 0.048 |  | 55.3% |  | 48.6% | 0.090 |
|  | Summer | 9.5 | 1.8 |  | 9.6 | 1.7 | 0.477 |  | 2.2 | 1.1 |  | 2.4 | 1.0 | 0.058 |  | 55.4% |  | 47.9% | 0.023 |
| 2023 | School | 10.5 | 1.8 |  | 10.6 | 1.5 | 0.672 |  | 2.2 | 1.1 |  | 2.4 | 1.1 | 0.038 |  | 50.4% |  | 47.9% | 0.526 |
|  | Summer | 10.8 | 1.6 |  | 10.7 | 1.6 | 0.528 |  | 2.3 | 1.0 |  | 2.5 | 1.0 | 0.236 |  | 51.1% |  | 47.4% | 0.316 |
